# Long non-coding RNA AL137789.1 promoted malignant biological behaviors and immune escape of pancreatic carcinoma cells

**DOI:** 10.1515/med-2023-0661

**Published:** 2023-04-01

**Authors:** Jing Wang, Yiyu Shen, Xiaoguang Wang, Zhongcheng Zhou, Zhengxiang Zhong, Tianyuan Gu, Bin Wu

**Affiliations:** Department of Hepatobiliary Surgery, The Second Affiliated Hospital of Jiaxing University, Jiaxing 314000, Zhejiang Province, China; Department of Hepatobiliary Surgery, The Second Affiliated Hospital of Jiaxing University, No. 397, Huancheng North Road, Jiaxing 314000, Zhejiang Province, China

**Keywords:** pancreatic carcinoma, long non-coding RNA AL137789.1, immune escape, proliferation, metastasis

## Abstract

Our pre-investigation has revealed that long non-coding RNA (LncRNA) AL137789.1 has the potential to predict the survival of patients with pancreatic carcinoma (PCa). Accordingly, the mechanism underlying the implication of AL137789.1 in PCa is covered in the current study. The non-tumor and paired tumor tissues were collected. Kaplan–Meier curve was employed to estimate the survival of PCa patients with high or low expression of AL137789.1. The proliferation, migration, invasion, and cell cycle of PCa cells were determined, and the cytotoxicity of CD8^+^ T cells was evaluated as well. Levels of AL137789.1, E-cadherin, N-cadherin, and Vimentin were quantified. According to the experimental results, AL137789.1 was highly expressed in PCa and related to a poor prognosis of patients. Overexpressed AL137789.1 enhanced the proliferation, migration, and invasion of PCa cells, increased the cell population at G2/M and S phases yet decreased that in G0/G1 phase, and diminished the cytotoxicity of CD8^+^ T cells. Also, overexpressed AL137789.1 elevated levels of N-cadherin and Vimentin, while lessening E-cadherin levels. However, the silencing of AL137789.1 produced contrary effects. Collectively, lncRNA AL137789.1 plays a tumor-promotive role in PCa by enhancing the progression and immune escape.

## 1 Introduction

Pancreatic carcinoma (PCa) is one of the deadliest and refractory malignancies, with a poor overall prognosis that has not improved over the past few decades [1]. In addition to many PCa cases being attributed to aging globally, several other key modifiable risk factors have been proposed, including tobacco consumption, obesity, diabetes mellitus, and alcohol intake [2]. Besides, inherited genetic factors, although they cannot be directly modified, are suggested to be pivotal risk factors for PCa, making it necessary to identify the genetic changes accounting for PCa, so as to deeply comprehend the etiology of PCa and provide early detection strategies [3].

Apart from the mutations or aberrant expressions of the protein-coding genes, the mutation and misregulation of non-coding RNAs, long non-coding RNAs (lncRNAs) in particular, play major roles in tumors [4]. LncRNAs, referring to the non-protein coding transcripts with over 200 nucleotides, possess a variety of functions like regulating gene expression, and they can therefore play essential roles in diverse biological functions and diseases including cancer [5,6]. LncRNA AGAP2 Antisense RNA 1 (AGAP2-AS1) is an lncRNA that enhances the proliferation and migration of PCa cells through epigenetically repressing the expressions of ankyrin repeat domain 1 (ANKRD1) and angiopoietin-like 4 (ANGPTL4) [7]. Inhibition of long intergenic non-protein coding RNA 994 (LINC00994) suppresses the malignant behaviors of PCa cells [8]. Similarly, lncRNA BM466146.1, also known as ZNFTR, exerts an anti-tumor effect on PCa [9].

Notably, novel immunotherapeutic strategies to induce a potent immune response against tumors, as well as the investigation of the molecular mechanism underlying the tumorigenesis of PCa, can not only offer some therapeutic opportunities for PCa patients but also contribute to optimizing treatment [10]. It has also been documented that clinically detected tumors inevitably evade immune responses during progressive growth, and the tumor cells in the process seem to exhibit immune escape from immune surveillance and even become the target of the tumor-antagonizing immune cells [11,12]. In terms of treating PCa, preclinical data have emphasized the significance of this immune evasion as the source of resistance to therapies, in which lncRNAs have been confirmed to participate as well [13,14]. Our pre-investigation has highlighted that lncRNA AL137789.1 (Ensembl ID: ENSG00000236911, Chromosome (GRCh38) 1: 207,551,925–207,606,555, DNA linear) has the potential to predict the survival of patients with PCa and is related to the tumor immune responses [15]. Herein, our study set out to extend the discovery regarding the role of AL137789.1 in PCa.

## 2 Methods

### 2.1 Specimen collection

The paired tumor and adjacent non-tumor tissues were obtained from the department of pathology in our hospital (*n* = 90 for each tissue), which were collected from the patients diagnosed with PCa and admitted in our hospital between January 2015 and October 2021. All tissues were rinsed with saline (B020; Nanjing Jiancheng Bioengineering Institute, Nanjing, China) and stored in liquid nitrogen until expression analysis.

### 2.2 *In situ* hybridization assay

The specimen was then subjected to *in situ* hybridization assay as previously reported [16]. In detail, the paraffin-embedded specimen was dewaxed in xylene (B50009; MERYER, Shanghai, China) and rehydrated using serial ethanol (from 100–25%, M34056; MERYER) for 5 min each time. Then, the specimen was immersed in diethylpyrocarbonate (DEPC)-treated phosphate buffer saline (PBS) for 5 min and digested with 20 mg/mL proteinase K (M38041; MERYER) at 37°C for 20 min. The specimen was acetylated in 0.25% (v/v) acetic anhydride (242845; Sigma-Aldrich, St Louis, MO, USA) in 0.1 M triethanolamine (pH 8.0, B70119; MERYER), prehybridized in 50% (v/v) deionized formamide (S4117; Sigma-Aldrich) in 2× SSC (M52512; MERYER) at 58°C for 1 hh, and hybridized with digoxin (DIG)-labeled probe of AL137789.1 (0.5 mg/mL, RiboBio, Guangzhou, China) in the hybridization buffer at 58°C overnight within a humidified chamber. Subsequently, the specimen was treated with RNase A (20 mg/mL, R6148; Sigma-Aldrich) at 37°C for 30 min and rinsed with 0.1× SSC at 58°C for 30 min. The hybridized probes were then detected with horseradish peroxidase (HRP)-conjugated anti-DIG antibody (DMABT-Z60500; Creative Diagnostics, Shirley, NY, USA) and stained with NBT/BCIP (B385716; Aladdin, Shanghai, China). The confocal laser scanning microscope (FV3000; Olympus, Tokyo, Japan) was used to observe the specimen at the magnification of ×200.

### 2.3 Cell culture

Human normal pancreatic duct cell line hTERT-HPNE (CRL-4023; ATCC, Manassas, Virginia, USA) was grown in Dulbecco’s modified Eagle’s medium (E600010; Sangon Biotech, Shanghai, China) with 5% fetal bovine serum (FBS, C0234; Beyotime, Shanghai, China), 10 ng/mL recombinant human EGF (P5552; Beyotime), 5.5 mM d-glucose (G7021; Sigma-Aldrich), and 750 ng/mL puromycin (ST551; Beyotime) as recommended by the producer. PCa cell lines BxPC3 (C1023), SW1990 (C1196), and PANC1 (C1004) were available from WHELAB (Shanghai, China). BxPC3 cells were maintained in RPMI-1640 medium (E600028; Sangon Biotech), SW1990 cells were cultured in F-12 basic medium (M0500; WHELAB), and PANC1 cells (C1004; WHELAB) were grown in the high-glucose DMEM (M0100; WHELAB). All media for PCa cells were blended with 10% FBS and 1% penicillin–streptomycin (G0100; WHELAB). Cell incubation was completed in an incubator (HF100; Heal Force, Shanghai, China) at 37°C with 5% CO_2_.

### 2.4 Cell transfection

As the expression of AL137789.1 was relatively higher in BxPC3 and PANC1 cells, these cells were employed for transfection. Specifically, BxPC3 and PANC1 cells at the density of 2 × 10^6^ cells per well were maintained in a six-well plate, and the cell confluence was pre-adjusted to 90%. The culture medium of cells was refreshed with 2 mL of fresh medium with FBS, and cells were transferred to a centrifuge tube containing 125 μL of Opti-MEM (11058-021; Gibco, Waltham, MA, USA) and plasmids including the overexpression or silence vector of AL137789.1 as well as their negative controls. Meanwhile, 5 μL of Lipo 6000 transfection reagent (C0526; Beyotime) was prepared in another tube. After being maintained at room temperature for 5 min, samples in these tubes were mixed together and incubated for 48 h. The overexpression vector of AL137789.1 was constructed by inserting the whole sequence into pcDNA 3.1 vector (V790-20; Invitrogen, Carlsbad, CA, USA), and an empty vector was used as the control. The silence vector of AL137789.1 (siAL137789.1 #1-#3, A01004) and its control (A06001) were acquired from GenePharma (Shanghai, China). The sequences used for transfection are listed in Table 1.

**Table 1:** Sequence for transfection

Gene	Target sequence (5′−3′)
si-AL137789.1 #1 sense oligo	UAACUGUUGCAGUUACUGCCU
si-AL137789.1 #1 antisense oligo	GCAGUAACUGCAACAGUUAAG
si-AL137789.1 #2 sense oligo	UUAACUGUUGCAGUUACUGCC
si-AL137789.1 #2 antisense oligo	CAGUAACUGCAACAGUUAAGA
si-AL137789.1 #3 sense oligo	UAAUCAAGAGGAAAAGUUCUU
si-AL137789.1 #3 antisense oligo	GAACUUUUCCUCUUGAUUAUC
si-NC sense oligo	UUAGUGCACUAUUACUGCCGU
si-NC antisense oligo	CAGGCUUAAGAAACAUAACUG

### 2.5 Cell viability assay

The viability of BxPC3 and PANC1 cells was evaluated by cell counting kit-8 (CCK-8) assay. In short, BxPC3 and PANC1 cells (2 × 10^3^ cells/well) were grown in the 96-well plates in the incubator at 37°C with 5% CO_2_ for 24, 48, and 72 h. Following the addition of 10 μL of CCK-8 reagent (C0037; Beyotime) for 4-h incubation, the optical density (OD) at 450 nm was read using a microplate reader (E1140; Beyotime).

### 2.6 Cell proliferation assay

Cell proliferation was detected via colony formation assay. Briefly, 1 × 10^3^ transfected BxPC3 and PANC1 cells were seeded in six-well plates for 14 days, and their culture medium was removed when the colonies formed were observable. For the visualization process, the colonies formed were fixed in 4% paraformaldehyde (PFA, P0099; Beyotime) for 10 min, dyed with crystal violet (C0221; Beyotime) for 1 min, and photographed with a Canon EOS 500D camera (Canon, Tokyo, Japan).

### 2.7 Migration and invasion assays

Cell migration and invasion were accessed through Transwell assay. In brief, 1 × 10^5^ transfected BxPC3 and PANC1 cells in the serum-free medium (100 μL) were inoculated onto the non-coated membrane (24-well insert with a pore size of 8 μm, 3422; Corning, Inc., Corning, NY, USA) or Matrigel-coated membrane (354234; Corning, Inc.) in the upper chamber [17]. Meanwhile, the lower chamber was supplemented with a basic culture medium with 10% FBS. After 48 h of incubation, cells passing through the membrane were fixed with 4% PFA and stained with 0.1% crystal violet for 5 min, while those failing to invade the membrane were carefully removed using a cotton swab. An inverted optical microscope (Eclipse Ni-U; Nikon, Tokyo, Japan) was used to observe the cells at the magnification of 250×.

### 2.8 Cell cycle assay

For cell cycle analysis, 1 × 10^6^ BxPC3 and PANC1 cells after transfection were harvested and fixed with cold 70% ethanol overnight. Next, cells were resuspended with 10 mg/mL RNase and 1 mg/mL propidium iodide (PI, M60251; MERYER) in PBS and incubated at room temperature for 30 min. BD FACS Calibur flow cytometer (BD Biosciences, San Diego, CA, USA) was used to access the DNA content, and the cell population (%) in different phases of the cell cycle was accessed using Cell Quest acquisition software (BD Biosciences) [18].

### 2.9 Isolation of peripheral blood monocular cells (PBMCs)

PBMCs were isolated from the blood samples of healthy donors using the Ficoll–Hypaque density gradient centrifugation [19]. Specifically, the blood sample collected was mixed with PBS, underlaid with Ficoll Paque^™^ PLUS (17144002; Cytiva, Marlborough, MA, USA), and centrifuged at 500 × *g* for 30 min. PBMCs were then harvested from the interface between the Ficoll and plasma layers, transferred to a new tube, and rinsed using 3× PBS supplemented with 5% FBS. The washed PBMCs were cryopreserved in 80% RPMI-1640 medium with 10% FBS and 10% dimethyl sulfoxide (DMSO, M23322; MERYER) in a freezing container (5100-0001; ThermoFisher Scientific, Waltham, MA, USA).

### 2.10 Preparation of CD8^+^ T cells

The CD8^+^ T cells were isolated from the PBMCs using Dynabeads^™^ CD8 (11333D; Invitrogen) as per the protocols. In detail, dynabeads were added into and co-incubated with the PBMCs (1 × 10^7^) within a tube at 4°C for 20 min. The tube was placed in a magnet for 2 min, and the supernatant was carefully removed. Thereafter, 1 mL of buffer 1 (including PBS (Ca^2+^- and Mg^2+^-free), 0.1% BSA (M29446; MERYER), and 2 mM EDTA (M20959; MERYER)) were added and pipetted five times in total. The cell pellet was resuspended in 100 μL of RPMI-1640 medium with 1% FBS and added with 10 μL of DETACHaBEAD at room temperature for 45 min. After the tube was placed in the magnet for 1 min again, the supernatant containing released cells was transferred to a new tube. The detached cells were washed via resuspension to 4 mL of RPMI-1640 medium with 1% FBS and centrifuged at 400 × *g* for 6 min to remove the DETACHaBEAD. The harvested cells were resuspended in RPMI-1640 medium with 1% FBS for subsequent analysis.

### 2.11 Lactate dehydrogenase (LDH) cytotoxicity assay

For LDH cytotoxicity assay, the CD8^+^ T cells (effector cell) isolated as described earlier were co-cultured with BxPC3 and PANC1 cells (target cell) at the indicated ratios of 2:1, 3:1, and 5:1 for 24 h [20]. The cytotoxicity of CD8^+^ T cells was evaluated by a commercial LDH cytotoxicity assay kit (C20300, Invitrogen). Briefly, the co-cultured cells were maintained in the 96-well plate in an incubator at 37°C with 5% CO_2_ overnight and added with 50 μL of prepared reaction mixture at room temperature for 30 min in the dark. Following the addition of 50 μL of stop solution, the absorbance at 490 nm was read using a microplate reader, and the cytotoxicity of cells was calculated using the formula [21]:

{\rm{Cytotoxicity}}( \% )=\left(1-\frac{{{\rm{OD}}}_{{\rm{case}}}-{{\rm{OD}}}_{{\rm{effector\; cell}}}}{{{\rm{OD}}}_{{\rm{target\; cell}}}}\right)\times 100 \% .]

### 2.12 RNA isolation and quantitative real-time PCR

TriZol (15596-018; Invitrogen) was used to extract total RNA from both tissues (tumor and non-tumor) and cells (hTERT-HPNE and PCa cells) as appropriate, and the RNA was preserved at −80°C for subsequent analysis. The synthesis of cDNA was carried out with a first-strand cDNA synthesis kit (K1621; ThermoFisher Scientific), and the primers for both AL137789.1 and housekeeping control GAPDH (Table 2) were synthesized as required. The quantitative PCR was conducted with SYBR Green PCR master mix (4309155; Applied Biosystems, Waltham, MA, USA) in 7500 real-time PCR system (4351107; Applied Biosystems) under the thermocycling conditions: 95°C for 10 min, 40 cycles of 95°C for 15 s and 60°C for 1 min, and the final extension at 72°C for 1 min. The expression level of AL137789.1 was normalized to that of GAPDH via the 2^−ΔΔCT^ method [22].

**Table 2:** Primer for quantitative reverse-transcription PCR

Gene	Forward Primer (5′−3′)	Reverse Primer (5′−3′)
AL137789.1	CCATATAATGATGAGGCAGT	AGGTCTTCTCTGACCAAATA
GAPDH	CCTCAACTACATGGTTTACA	TGTTGTCATACTTCTCATGG

### 2.13 Protein extraction and Western blot

Transfected cells were collected and lysed in RIPA lysis buffer (M52297; MERYER) to harvest the total protein; the concentration of which was determined by a BCA protein assay kit (M52286; MERYER). About 30 μg protein sample was detached with SDS-PAGE (M52283; MERYER) and transferred to PVDF membrane (FFP33; Beyotime), following which 5% defatted milk was used to block the membrane. The primary antibodies against E-cadherin (ab231303, 1:2,000, 97 kDa), N-cadherin (ab76011, 1:5,000, 100 kDa), Vimentin (ab8978, 1:2,000, 53 kDa), and housekeeping gene GAPDH (ab181602, 1:10,000, 36 kDa) were co-incubated with the membrane at 4°C overnight. After washing, the membrane was cultured with the secondary antibodies horseradish peroxidase (HRP)-conjugated goat anti-rabbit IgG (1:1,000, A0208; Beyotime) and goat anti-mouse IgG (1:1,000, A0216; Beyotime) at room temperature for 1 h. ECL Western blotting substrate (M52285; MERYER) was used to visualize the immunoreactivity, and the protein was quantified via Quantity One software (Bio-Rad, Hercules, CA, USA) [21].

### 2.14 Statistical analysis

All data from three independent tests were analyzed using GraphPad 8 (GraphPad, Inc., La Jolla, CA, USA) and expressed as mean ± standard deviation. The recruited patients with PCa in our hospital were allocated to the high or low AL137789.1 group (*n* = 60 for low AL137789.1 group and *n* = 30 for high AL137789.1 group). The correlation between AL137789.1 expression and the survival time of patients, which was monitored every 20 months (for 80 months in total), was analyzed via Kaplan–Meier curve and a log-rank test. The expression level of lncRNA AL137789.1 in paired tumor and non-tumor tissues was compared using paired Student’s *t*-test. One-way ANOVA showed significant differences among groups. Kaplan–Meier curve with log-rank test was used to analyze the survival of patients with PCa. The statistical significance was deemed when the *p*-value was below 0.05.


**Ethics statement:** The Ethics Committee of the Second Affiliated Hospital of Jiaxing University has carefully reviewed and approved the conduction of the current study (ethic endorse no. JXEY-2019JX144). All patients or their guardians have been informed of and agreed on the usage of the tissue in our research.

## 3 Results

### 3.1 LncRNA AL137789.1 was highly expressed in PCa and associated with poor survival of patients with PCa

**Figure 1:**
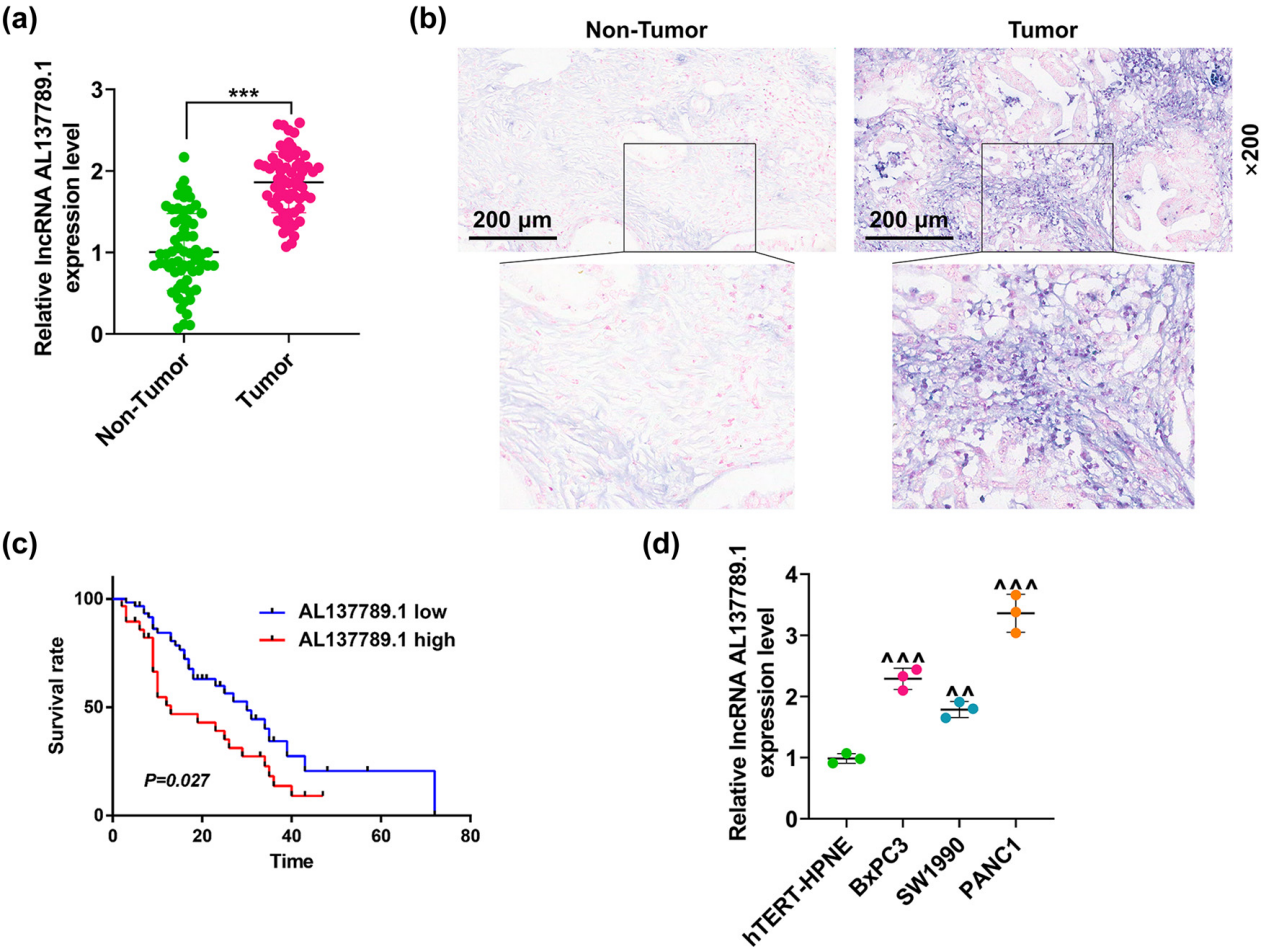
AL137789.1 was highly expressed in PCa and associated with a poor prognosis in patients with PCa. (a) AL137789.1 expression level in paired tumor (PCa) and non-tumor tissues (*n* = 90 for each tissue), as reflected by the results of quantitative real-time PCR. (b) The expression of AL137789.1 in tumor (PCa) and non-tumor tissues based on the results of *in situ* hybridization. Magnification: ×200, scale bar = 200 μm. (c) Survival analyses on PCa patients with high or low AL137789.1 levels based on the Kaplan–Meier curve with log-rank test (*n* = 60 for low AL137789.1 group and *n* = 30 for high AL137789.1 group). (d) Evaluation on AL137789.1 expression level in pancreatic duct cell line hTERT-HPNE and PCa cells (BxPC3, SW1990, and PANC1) by quantitative real-time PCR, *n* = 3. ^^^^ *p* < 0.01, ^***^ or ^^^^^ or ^+++^ *p* < 0.001. ^*^vs non-tumor; ^^^vs hTERT-HPNE. Abbreviation: PCa: pancreatic cancer.

**Figure A1:**
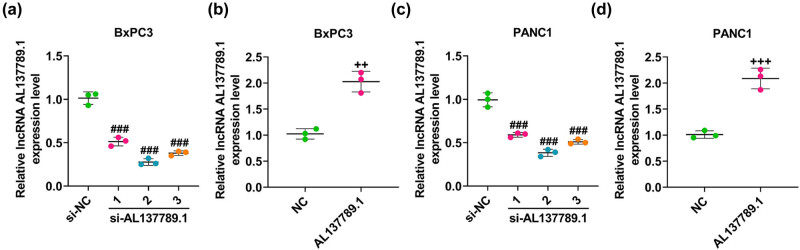
AL137789.1 was highly expressed in PCa and associated with a poor prognosis in patients with PCa. (a) AL137789.1 expression level in paired tumor (PCa) and non-tumor tissues (*n* = 90 for each tissue), as reflected by the results of quantitative real-time PCR. (b) The expression of AL137789.1 in tumor (PCa) and non-tumor tissues based on the results of *in situ* hybridization. Magnification: ×200, scale bar = 200 μm. (c) Survival analyses on PCa patients with high or low AL137789.1 levels based on the Kaplan–Meier curve with log-rank test (*n* = 60 for low AL137789.1 group and *n* = 30 for high AL137789.1 group). (d) Evaluation on AL137789.1 expression level in pancreatic duct cell line hTERT-HPNE and PCa cells (BxPC3, SW1990, and PANC1) by quantitative real-time PCR, *n* = 3. ^^^^ *p* < 0.01, ^***^ or ^^^^^ or ^+++^ *p* < 0.001. ^*^vs non-tumor; ^^^vs hTERT-HPNE. Abbreviation: PCa: pancreatic cancer.

The paired tumor (PCa) and non-tumor (normal) tissues were collected to quantify the level of AL137789.1, and an increased expression of AL137789.1 was observed in paired tumor tissue (Figure 1a, *p* < 0.001), which was further confirmed through *in situ* hybridization (Figure 1b). Also, the survival analysis suggested that a higher AL137789.1 level was associated with a poor survival of patients with PCa (Figure 1c, *p* = 0.027). Meanwhile, the level of AL137789.1 was quantified in normal pancreatic duct cells (hTERT-HPNE) and PCa cells (BxPC3, SW1990, and PANC1). The comparison results demonstrated the level of AL137789.1 was relatively higher in PCa cells BxPC3 and PANC1 (Figure 1d, *p* < 0.01). As such, these two cells were used for subsequent analyses and transfected with si-AL137789.1 #1-#3 and the overexpression plasmid of AL137789.1 as needed. The higher or lower expression of AL137789.1 in these cells proved that the transfection was successful (Figure A1a–d, *p* < 0.01), and si-AL137789.1#2 was selected for our study with the highest knock-down efficiency.

### 3.2 LncRNA AL137789.1 affected the cell viability, colony formation, and cell cycle of PCa cells

**Figure 2:**
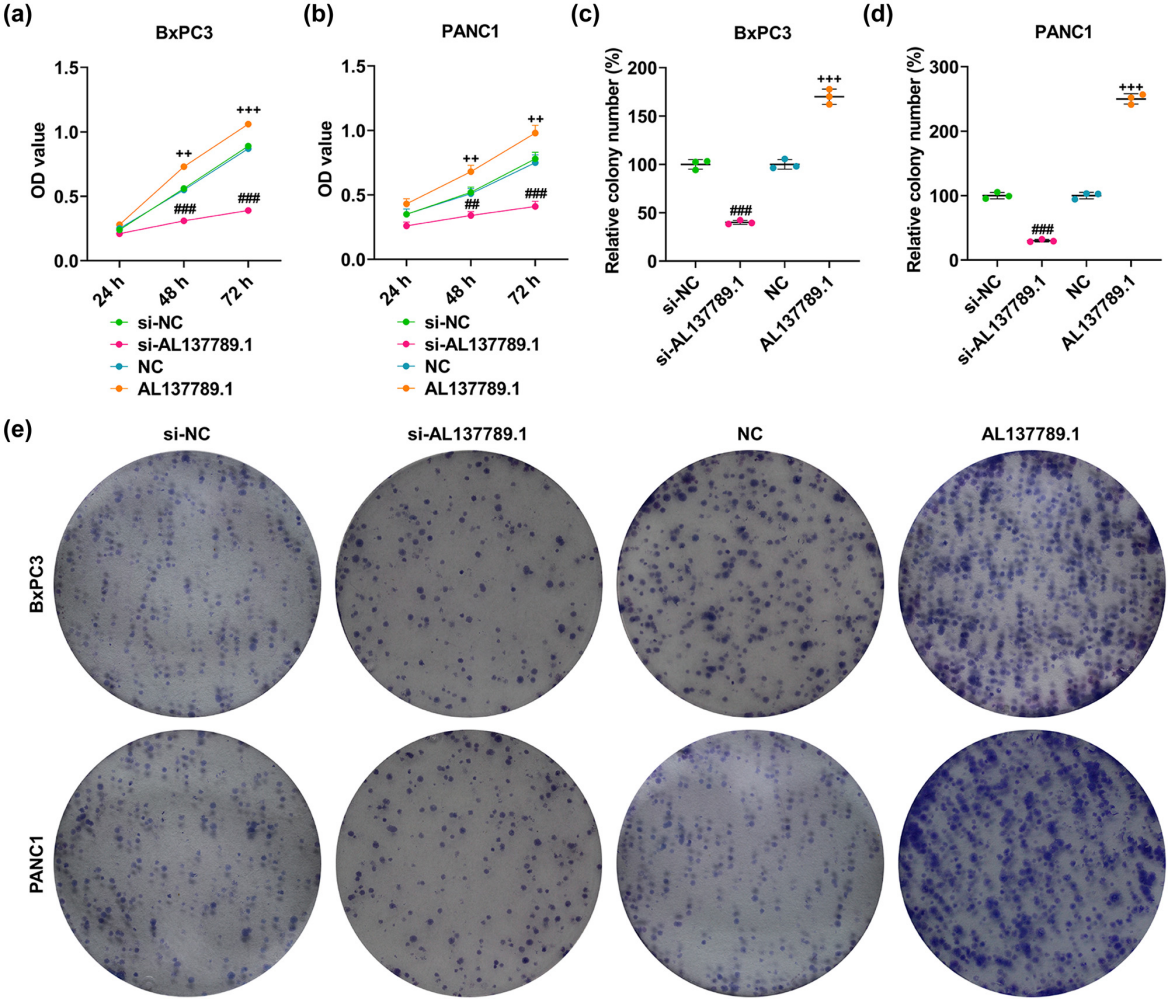
Role of AL137789.1 in the viability and colony formation of PCa cells. (a–b) The OD value of PCa cells BxPC3 and PANC1 at 24, 48, and 72 h following the overexpression or silencing of AL137789.1 according to the results of the CCK-8 assay. (c–e) The quantified number of colonies formed in PCa cells BxPC3 and PANC1 following the overexpression or silencing of AL137789.1 based on the results of the colony formation assay. All data from three independent tests were expressed as mean ± standard deviation. ^##^ or ^++^ *p* < 0.01, ^###^ or ^+++^ *p* < 0.001. ^#^vs si-NC; ^+^vs NC. Abbreviation: PCa: pancreatic cancer; si-RNA; small interfering RNA; NC: negative control; OD: optical density; h: hour; CCK-8: cell counting kit-8.

**Figure 3:**
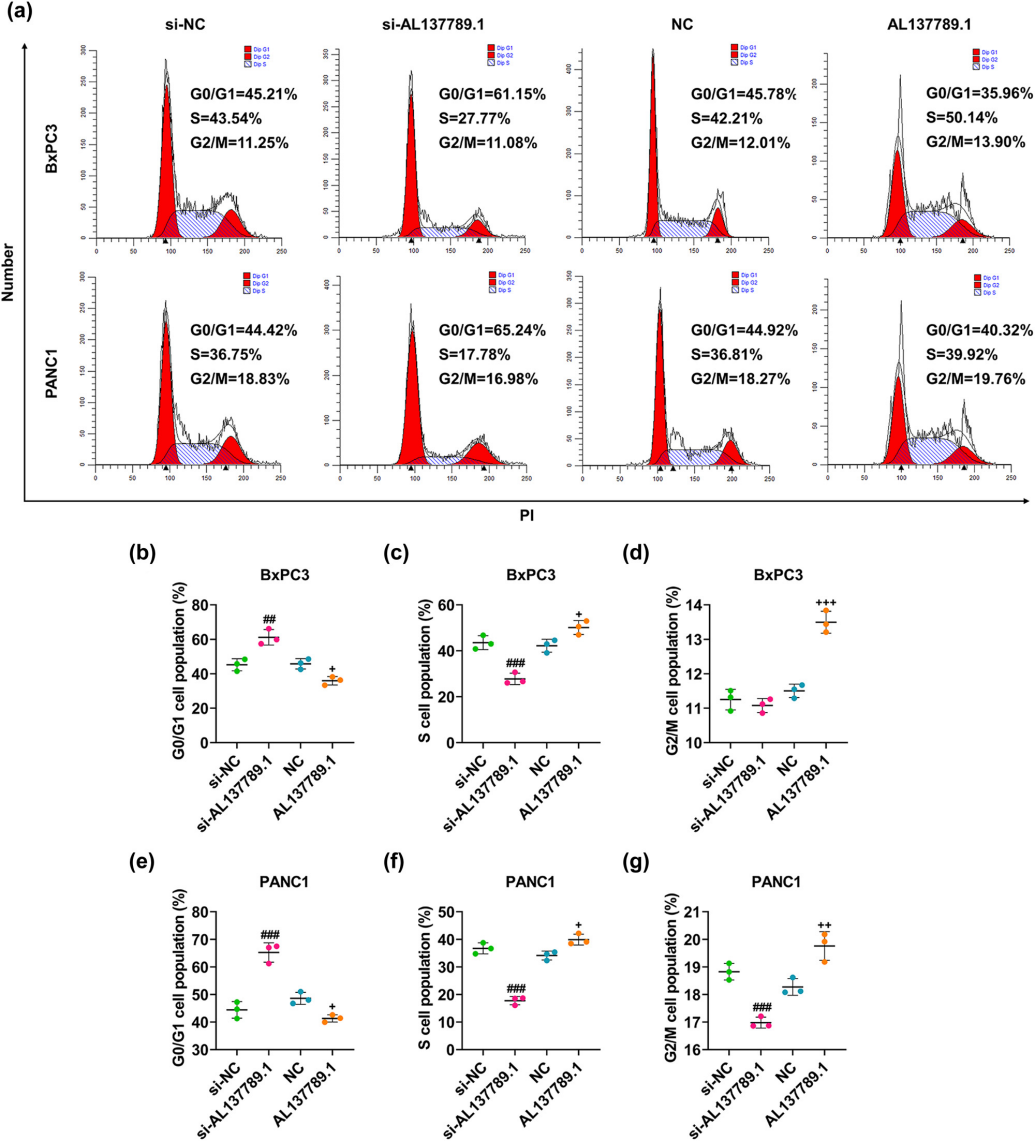
Regulation of AL137789.1 on the cell cycle of PCa cells. (a) Visualization on cell population at G0/G1, S and G2/M phases of (b–d) BxPC3 and (e–g) PANC1 cells following the overexpression or silencing of AL137789.1 using flow cytometry. All data from three independent tests were expressed as mean ± standard deviation. ^#^ or ^+^ *p* < 0.05, ^##^ or ^++^ *p* < 0.01, ^###^ or ^+++^ *p* < 0.001. ^#^ vs si-NC; ^+^ vs NC. Abbreviation: PCa: pancreatic cancer; si-RNA; small interfering RNA; NC: negative control.

In accordance with the results, AL137789.1 silencing could evidently decrease the OD value at 48 and 72 h (Figure 2a and b, *p* < 0.01), and the number of colonies formed in BxPC3 and PANC1 cells (Figure 2c and d, *p* < 0.001). On the contrary, AL137789.1 overexpression increased the OD value at 48 and 72 h (Figure 2a and b, *p* < 0.01), and the number of colonies formed (Figure 2c and d, *p* < 0.001). In addition, the silencing of AL137789.1 increased the population of cells at G0/G1 phase, yet decreased the percentage of cells at S and G2/M phases (Figure 3a–g, *p* < 0.01). However, AL137789.1 overexpression caused opposite results (Figure 3a–g, *p* < 0.05).

### 3.3 LncRNA AL137789.1 impacted the migration, invasion, and epithelial-to-mesenchymal formation of PCa cells and the resistance to CD8^+^ T cells

**Figure 4:**
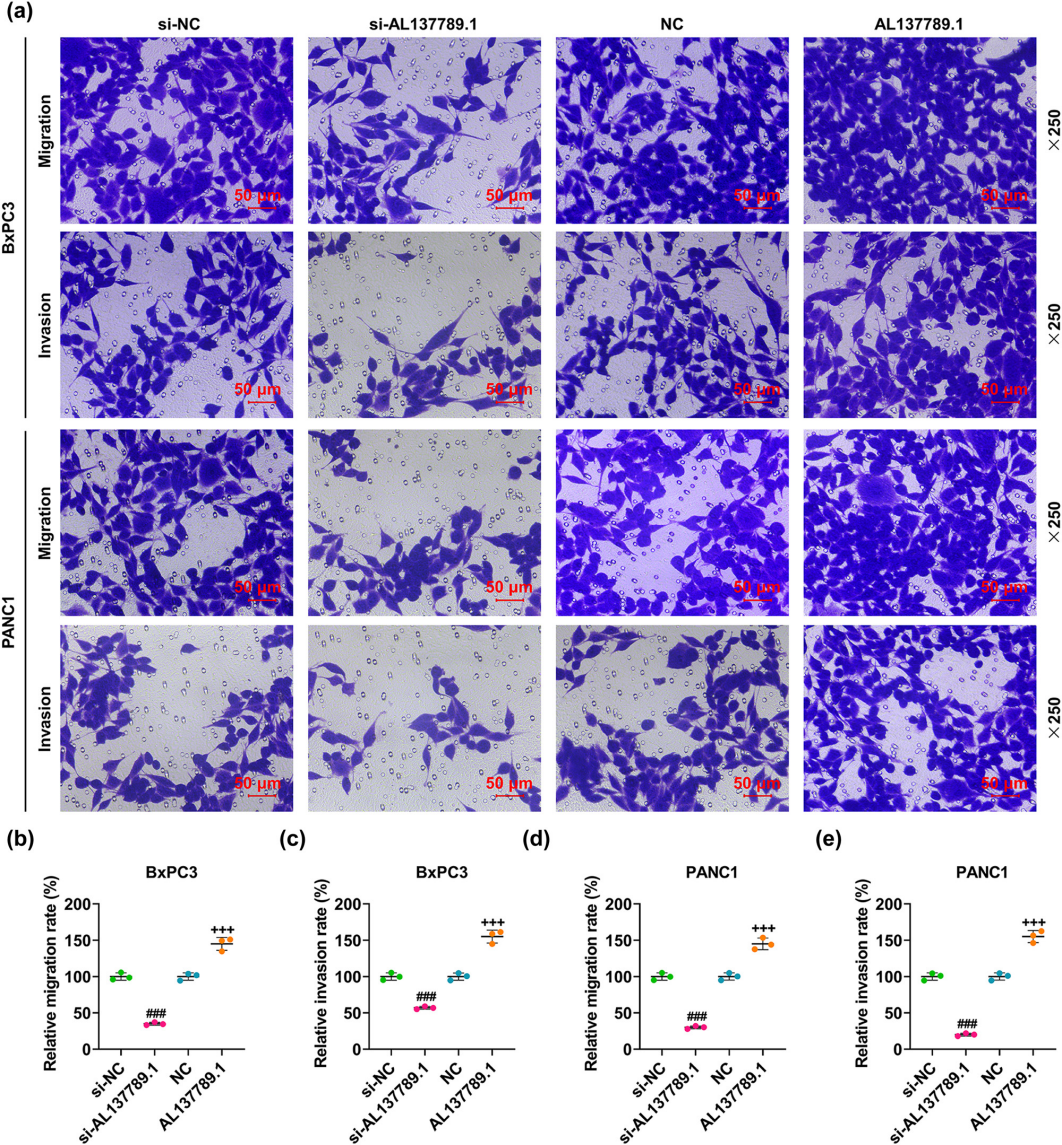
Effects of AL137789.1 on the migration and invasion of PCa cells. (a) The Transwell assay showed the images of migration and invasion of PCa cells BxPC3 and PANC1 at 48 h following the overexpression or silencing of AL137789.1. (b–e) The quantification of migration and invasion rates of PCa cells BxPC3 and PANC1 at 48 h following the overexpression or silencing of AL137789.1 in accordance with the results of Transwell assay. Magnification: ×250; Scale bar = 50 μm. All data from three independent tests were expressed as mean ± standard deviation. ^###^ or ^+++^ *p* < 0.001. ^#^ vs si-NC; ^+^vs NC. Abbreviation: PCa: pancreatic cancer; si-RNA; small interfering RNA; NC: negative control; h: hour.

**Figure 5:**
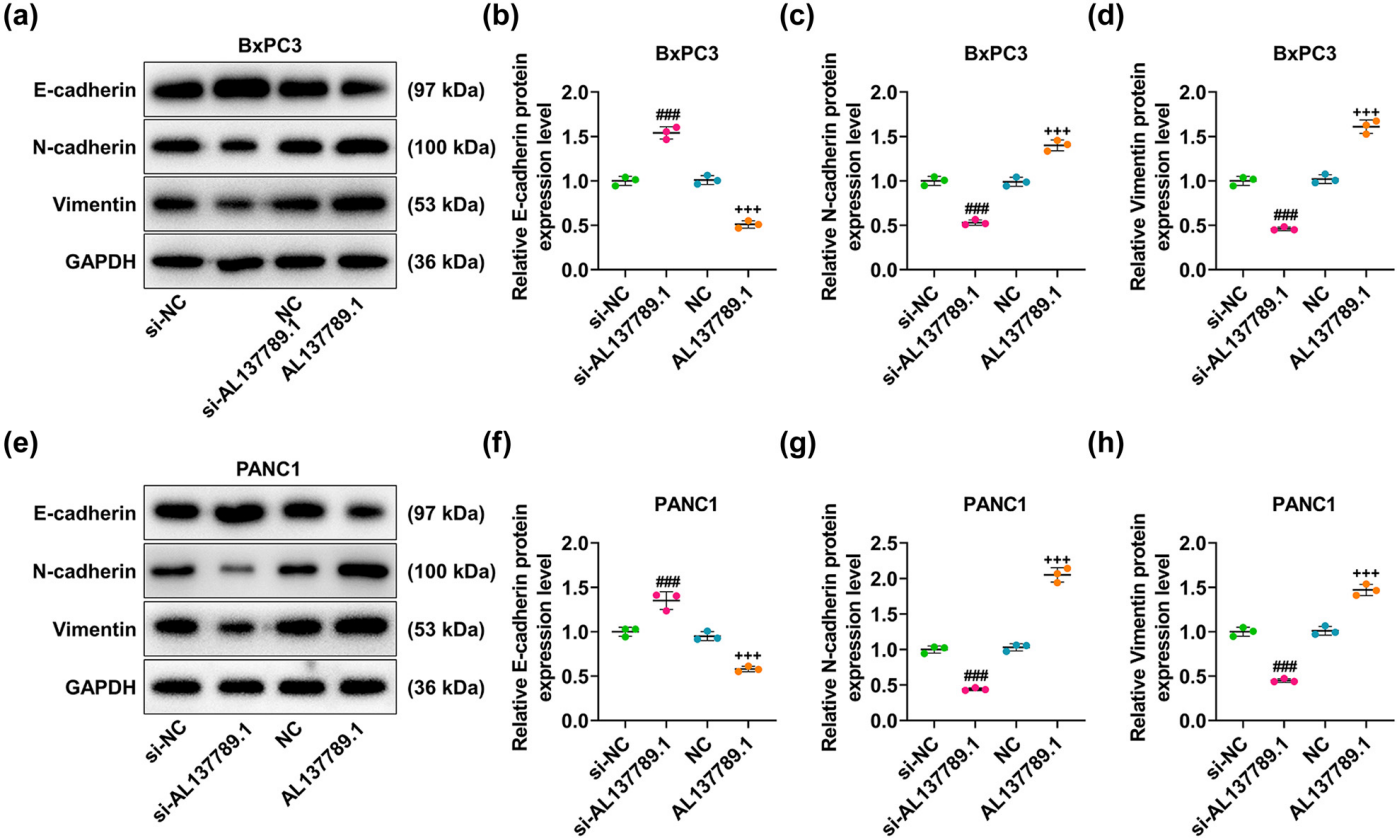
Modulation of AL137789.1 on the molecular markers in PCa cells. (a–h) The calculation of the protein expressions of molecular markers related to cell migration and invasion (E-cadherin, N-cadherin, and Vimentin) using Western blot. GAPDH was used as the housekeeping gene. All data from three independent tests were expressed as mean ± standard deviation. ^###^ or ^+++^ *p* < 0.001. ^#^ vs si-NC; ^+^vs NC.

The migration and invasion rates of BxPC3 and PANC1 cells were decreased by AL137789.1 silencing (Figure 4a–e, *p* < 0.001), but increased by AL137789.1 overexpression (Figure 4a–e, *p* < 0.001). The molecular markers related to the migration and invasion (E-cadherin, N-cadherin, and Vimentin) were further measured using Western blot assay. As can be noticed from the results, AL137789.1 silencing elevated the protein expression of E-cadherin yet diminished those of N-cadherin and Vimentin in PCa cells BxPC3 and PANC1 (Figure 5a–h, *p* < 0.001), while overexpression of AL137789.1 promoted the protein expressions of N-cadherin and Vimentin but repressed that of E-cadherin (Figure 5a–h, *p* < 0.001).

### 3.4 LncRNA AL137789.1 affected the cytotoxicity of CD8^+^ T cells to PCa cells

**Figure 6:**
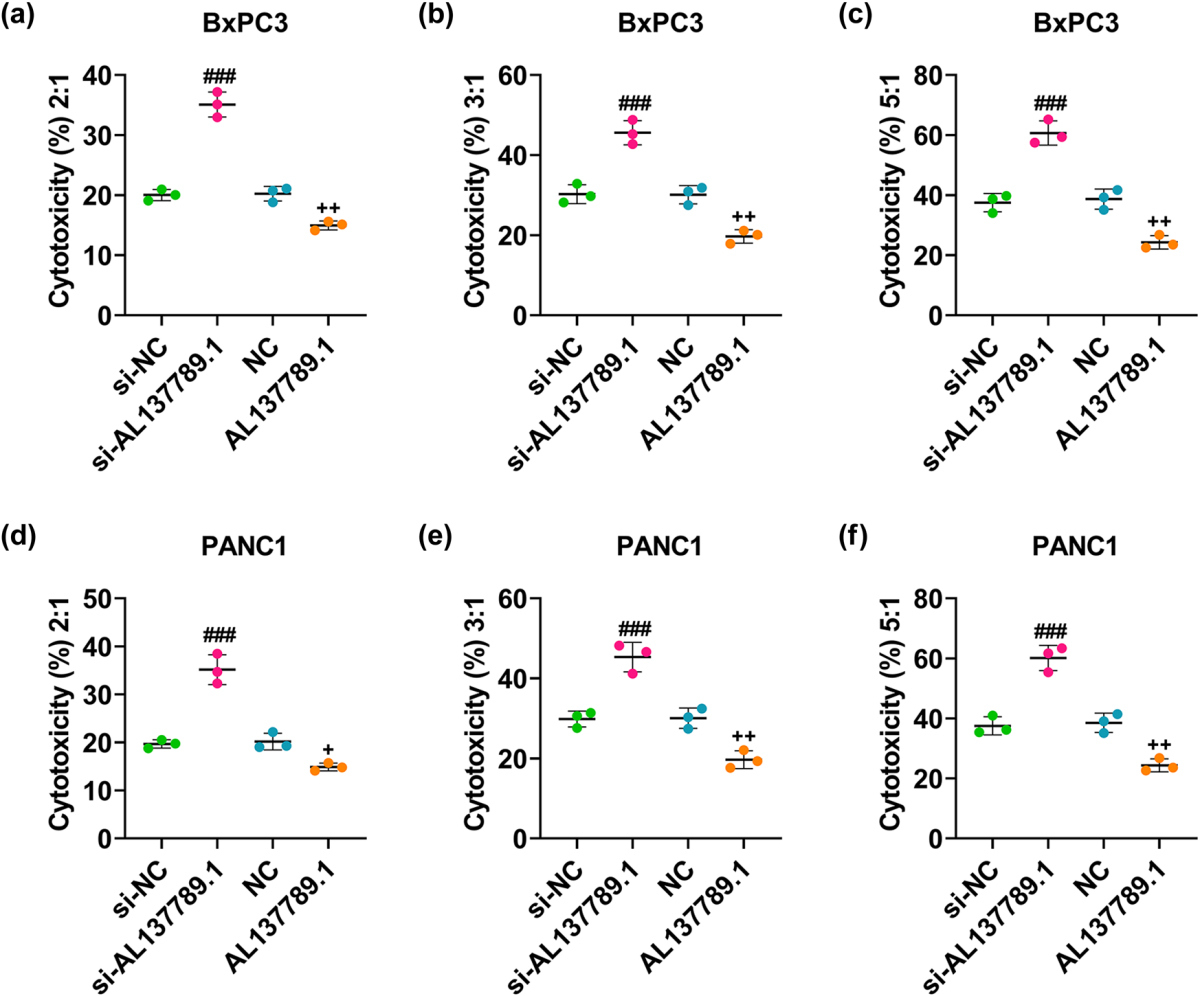
Modulation of AL137789.1 on the immune escape in PCa cells. (a–f) The cytotoxicity of CD8^+^ T cells to PCa cells BxPC3 and PANC1 measured based on the LDH cytotoxicity test. All data from three independent tests were expressed as mean ± standard deviation. + *p* < 0.05, ^++^ *p* < 0.01, ^###^ *p* < 0.001. ^#^ vs si-NC; ^+^vs NC.

The cytotoxicity of CD8^+^ T cells to PCa cells was increased following the silencing of AL137789.1 (Figure 6a–f, *p* < 0.001). However, the overexpression of AL137789.1 in these cells reduced the cytotoxicity of CD8^+^ T cells to PCa cells (Figure 6a–f, *p* < 0.05).

## 4 Discussion

Here, combined with our pre-investigation on the role of lncRNA AL137789.1 in PCa [15], we additionally proved that lncRNA AL137789.1, which was highly expressed in PCa, could promote the proliferation, migration, invasion, and immune escape yet inhibit cell cycle arrest in PCa cells. These findings, to some extent, further refined our research and knowledge of the biological role of lncRNA AL137789.1 in PCa.

In PCa cells, proliferation plays a pivotal part in the development and progression of tumors, and the deregulation of the migration and invasion causes tumor cells to migrate from the primary tumors and invade the adjacent normal tissues [23,24,25]. Meanwhile, the interaction between PCa cells and stromal cells has been documented to induce the tumor microenvironment, the components of which not only promote metastasis via stimulating migration/invasion and EMT but also contribute to immunosuppression [26]. E-Cadherin and N-cadherin are two members of the cadherin family (the protein of the junctional complex). Of them, E-cadherin is crucial to maintain the epithelial state and the integrity of the epithelial barrier, and N-cadherin is an essential molecule that affects the EMT and is related to aggressive tumor behavior [27,28]. Vimentin has been suggested as a marker of EMT, and the hallmarks of EMT also include both the loss of E-cadherin and the acquisition of mesenchymal marker N-cadherin [29,30]. Accumulating research has highlighted the role and participation of lncRNAs in modulating proliferation, migration, and invasion of tumor cells [31,32,33,34]. The modulation of lncRNAs on the proliferation, migration, invasion, and cell cycle of PCa cells as well as the levels of these markers has been systematically investigated and interpreted [35,36,37]. Accordingly, we wondered if AL137789.1 could play a similar role. The results in the current research confirmed the promotive effects of AL137789.1 overexpression on the proliferation, migration, and invasion and its inhibitory effect on the cell cycle of PCa cells (BxPC3 and PANC1), along with reduced expression of E-cadherin and increased expressions of N-cadherin and Vimentin, which enriched the role of AL137789.1 in PCa.

More importantly, AL137789.1 is a component of the lncRNA signature which is related to the tumor immune responses and established to predict the survival of patients with PCa based on our pre-investigation [15]. Our study verified that AL137789.1 reduced the cytotoxicity of CD8^+^ T cells to PCa cells, so as to enhance the immune escape of PCa cells. The activation of cancer-specific T cells kills tumor cells through the recognition of tumor-specific antigens [38]. As a key anti-tumor immune component of cancer-specific T cells, CD8^+^ T cells contact physically with malignant tumor cells and cause the death of tumor cells by activating their intracellular signals during tumor immunity [39,40]. In other words, CD8^+^ T cells infiltrate the core or invading site of the tumor and play critical roles in killing cancer cells [40]. In the occurrence of immune escape, CD8^+^ T cells are excluded from the vicinity of cancer cells with inhibited immune function, leading to the lack of CD8^+^ T cells in the tumor microenvironment [41,42,43]. Tumor immune escape is an important part of tumorigenesis and development, during which tumor cells develop various immunosuppressive mechanisms to combat tumor immunity [44]. The selection of immunoresistant tumor cells and the abnormality in antitumor-related immune cells like T cells have been proposed as the mechanism of tumor immune escape [45]. So far, the research on the roles of lncRNAs in the immune escape of PCa is limited, with only one suggestion that ncRNAs can be distributed by small extracellular vesicles (sEVs) in PCa, thereby facilitating the intracellular communication and affecting the cancer hallmarks including angiogenesis, immune escape, and metastatic dissemination [46]. Herein, we newly identified the effects of AL137789.1 on the immune escape of PCa cells; i.e., the overexpression of AL137789.1 reduced the cytotoxicity of CD8^+^ T cells to PCa cells and thus promoted the immune escape within these tumor cells.

It is well-known that lncRNA could act as a competing endogenous RNA (ceRNA), thereby competing for posttranscriptional control by sponging certain relevant microRNAs (miRNAs) [47]. For example, lncRNA SLCO4A1-AS1 knockdown inhibits growth, migration, and invasion, and induces apoptosis of PCa cells by acting as a ceRNA to modulate miR-4673/KIF21B axis [34]; lncRNA HCG11/miR-579-3p/MDM2 axis regulates malignant biological properties in PCa cells [48]. Considering that ceRNA is a known mechanism of lncRNA, future studies will further deeply expound the mechanism of ceRNA of AL137789.1 in PCa. Besides, since all these results are solely concluded based on the *in vitro* assays, *in vivo* assays will be carried out in the future to validate these results, and other lncRNAs that might participate in PCa are also worth studying.

Collectively, our current study interprets the role and effects of AL137789.1, an lncRNA highly expressed in PCa and a component of the lncRNA signature that is related to the tumor immune responses and established to predict the survival of patients with PCa, which could accelerate the proliferation, migration, invasion, and cell cycle, and promote the immune escape of PCa cells BxPC3 and PANC1.
